# Supplementary figures and images for: Magnitude and factors associated with post-tuberculosis lung disease in low- and middle-income countries: A systematic review and meta-analysis
Source: PLOS Glob Public Health. 2022 Dec 20;2(12):e0000805. doi: 10.1371/journal.pgph.0000805 (PMC10021795; doi:10.1371/journal.pgph.0000805)

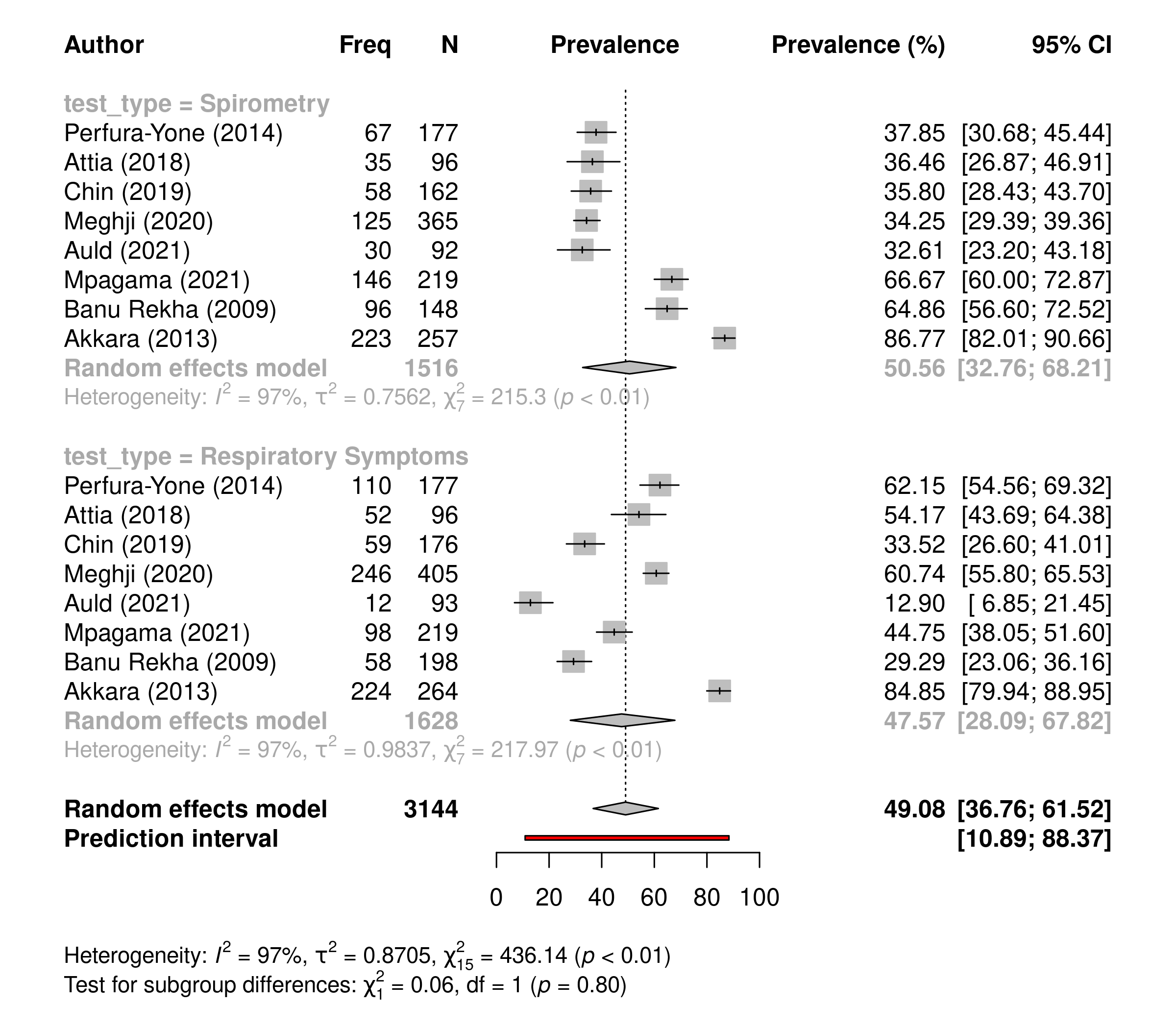

Supplement: S1 Fig — (TIF) [file pgph.0000805.s001.tif]

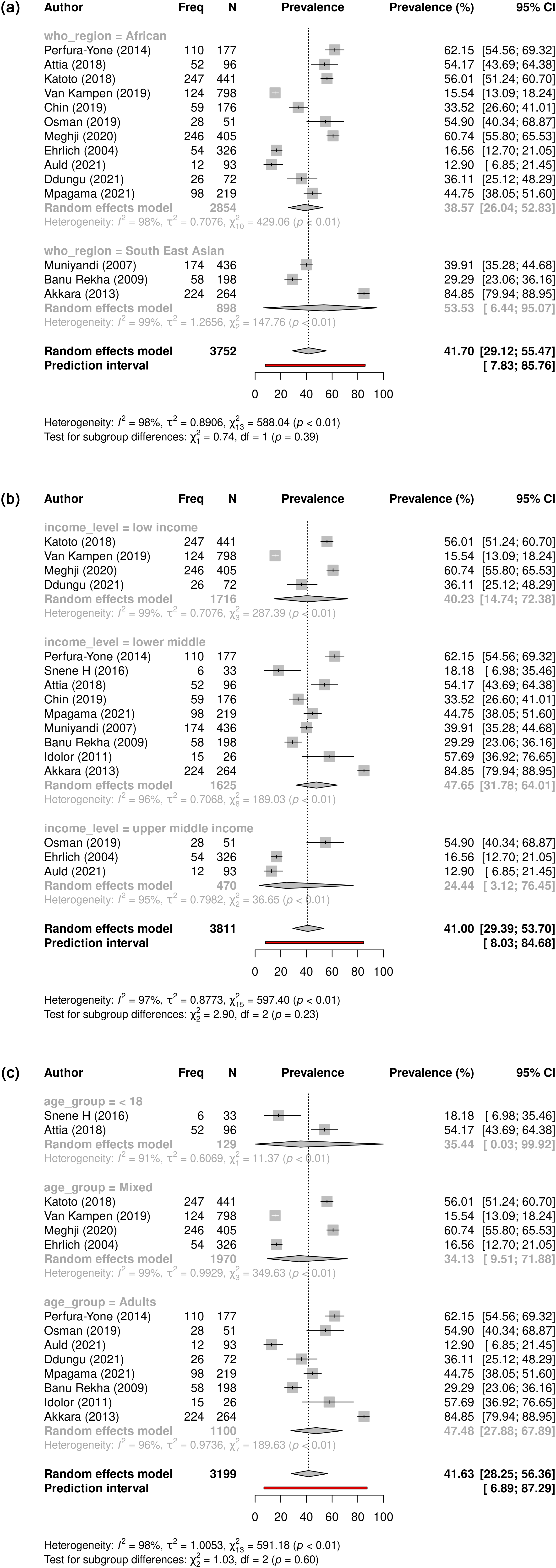

Supplement: S2 Fig — a. Prevalence of persistent respiratory symptoms across WHO regions. b. Prevalence of persistent respiratory symptoms across income levels. c. Prevalence of persistent respiratory symptoms across age groups. (TIF) [file pgph.0000805.s002.tif]

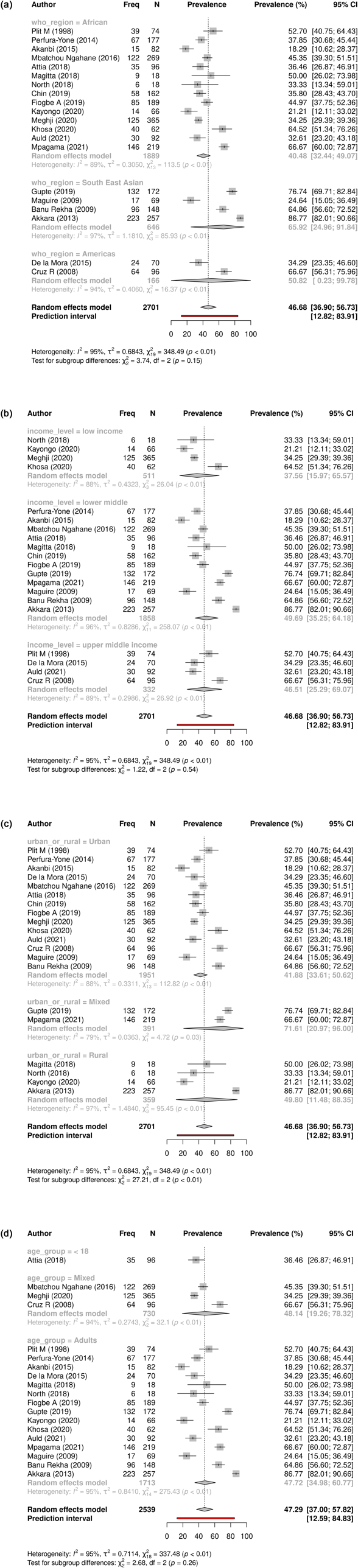

Supplement: S3 Fig — a. Prevalence of abnormal spirometry across WHO regions. b. Prevalence of abnormal spirometry across income levels. c. Prevalence of abnormal spirometry in rural, mixed, and urban settings. d. Prevalence of abnormal spirometry across age groups. (TIF) [file pgph.0000805.s003.tif]

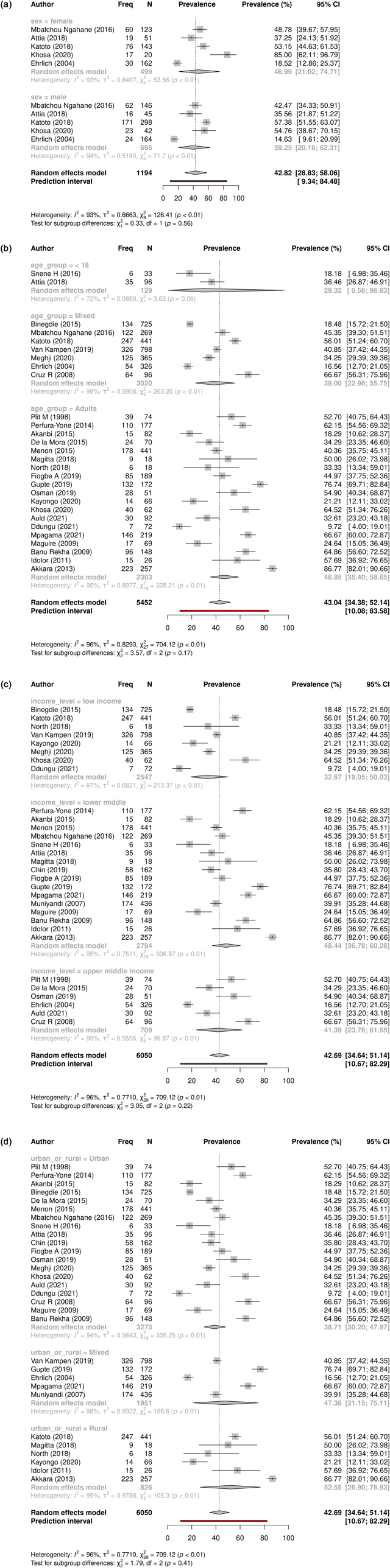

Supplement: S4 Fig — a. Prevalence of PTLD as a composite outcome in female and male subgroups. b. Prevalence of PTLD as a composite outcome across age groups. c. Prevalence of PTLD as a composite outcome across income levels. d. Prevalence of PTLD as a composite outcome in urban, rural, and mixed settings. (TIF) [file pgph.0000805.s004.tif]
