# Supplementary material for: Magnitude and factors associated with post-tuberculosis lung disease in low- and middle-income countries: A systematic review and meta-analysis
Source: PLOS Glob Public Health. 2022 Dec 20;2(12):e0000805. doi: 10.1371/journal.pgph.0000805 (PMC10021795; doi:10.1371/journal.pgph.0000805)
Supplement: S1 Table — (PDF) [file pgph.0000805.s005.pdf]

Supporting Table 1: Search terms used for systematic review on post-tuberculosis lung disease in low- and middle-income countries.

Estimate	Magnitude	Post Tuberculosis	Lung disease	LMICs / Resource limited settings
Approximate	Prevalence	Post TB	Lung sequelae	Low-income countries
Calculate	Incidence	Post pulmonary tuberculosis	Chronic lung disease	Lower-middle-income countries
Compute	Occurrence	Post infectious	Lung dysfunction	Upper-middle-income countries
Determine	Extent	Pulmonary TB sequelae	Lung injury	Sub-Saharan Africa
Evaluate	Frequency	Pulmonary tuberculosis sequelae	Restrictive lung disease	Africa
Quantify		Tuberculosis sequelae	Lung function impairment	East Asia
Assess		Intrathoracic TB	Lung fibrosis	South Asia
Measure			Interstitial lung disease	Middle East
			Cavitation	South America
			Abnormal spirometry	Latin America
			Airflow obstruction	Resource-limited
			Bronchiectasis	South-East Asia
			Chronic obstructive pulmonary disease	
			COPD	
			Bronchiolitis obliterans	
			Obliterative bronchiolitis	
			Aspergillosis	
			Obstructive lung disease	
			Residual damage	
			Sequelae	
			Abnormal pulmonary function	
			Lung impairment	
			Impaired lung function	
			Abnormal spirometry	
			Spirometry	
			Airflow obstruction	
			Lung damage	
			Chronic bronchitis	
			Lung cavitation, cavities	
