# Supplementary material for: Magnitude and factors associated with post-tuberculosis lung disease in low- and middle-income countries: A systematic review and meta-analysis
Source: PLOS Glob Public Health. 2022 Dec 20;2(12):e0000805. doi: 10.1371/journal.pgph.0000805 (PMC10021795; doi:10.1371/journal.pgph.0000805)
Supplement: Attachment — Submitted filename: Rebuttal response letter_PLOS GH_Maleche-Obimbo_09aug2022.pdf [file pgph.0000805.s007.pdf]

UNIVERSITY OF NAIROBI
FACULTY OF HEALTH SCIENCES
SCHOOL OF MEDICINE
DEPARTMENT OF PAEDIATRICS AND CHILD HEALTH

Telephone: +(254) 20 4915046
Email : dept-paediatrics@uonbi.ac.ke

Kenyatta National Hospital
P.O. Box 19676
Nairobi, Kenya. 00202

To:
The Editor- in-chief,
PLoS Global Public Health

9th August 2022

Dear Madam,

RE: Response to Reviewers: Magnitude and factors associated with post-tuberculosis lung disease in low- and middle-income settings: a systematic review and meta-analysis (PGPH-D-22-01006)

Thank you very much for reviewing our above-mentioned systematic review and for giving us an opportunity to address the reviewers' comments. The comments were helpful and valuable to improve and refine the manuscript. We have revised the manuscript accordingly and submit both track changes and clean versions of the revised manuscript. We have made a point-by-point response to the comments of both the editor and the reviewers, each response is inserted below the specific comment. All the required documents have been submitted using the online system.

We do hope that we have responded adequately to the comments of the reviewers, and that we have addressed all the requirements of the editors and journal. Once again, we thank PLOS Global Public Health and the reviewers for your timely review of this systematic review and meta-analysis on post-tuberculous lung disease in low- and middle-income countries.

We look forward to hearing from you.

Yours Sincerely,

Elizabeth Maleche-Obimbo
Professor of Paediatrics & Respiratory Medicine
Epidemiologist and Paediatric Pulmonologist
Department of Paediatrics & Child Health
Faculty of Medicine, University of Nairobi.
Email: Elizabeth.obimbo@uonbi.ac.ke

<u>Response to Reviewers Comments:</u>

Journal Requirements:

1. Please amend your online Financial Disclosure statement. If you did not receive any funding for this study, please simply state: "The authors received no specific funding for this work."

   Response: The authors received no specific funding for this work. We have amended the Financial Disclosure statements as advised

2. Please update your online Competing Interests statement. If you have no competing interests to declare, please state: "The authors have declared that no competing interests exist.

   Response: The authors declare that no competing interests exist. We have amended the Competing Interest statement as advised.

3. In the online submission form, *you indicated that your data will be submitted to a repository upon acceptance.* We strongly recommend all authors deposit their data before acceptance, as the process can be lengthy and hold up publication timelines. Please note that, though access restrictions are acceptable now, your entire data will need to be made freely accessible if your manuscript is accepted for publication. This policy applies to all data except where public deposition would breach compliance with the protocol approved by your research ethics board. If you are unable to adhere to our open data policy, please kindly revise your statement to explain your reasoning and we will seek the editor's input on an exemption. Please be assured that, once you have provided your new statement, the assessment of your exemption will not hold up the peer review process.

   Response: We revise our statement regarding provision of data. The data has been submitted to the journal together with the revised manuscript. (We do not have access to a secure data repository in our institution, and on realising that there is an option for us to submit the data to the journal we opt to do so, and do hope that this fulfils the journal requirements).

4. We ask that a manuscript source file is provided at Revision. Please upload your manuscript file as a .doc, .docx, or .rtf.

   Response: We have submitted a marked-up copy of the manuscript that highlights changes made, and also submitted a clean version.

5. Please update your *Supporting Information list of legends to include all supplementary files.*

Response: We have updated the supporting information list of legends to include all supplementary files.

6. Please review your reference list to ensure that it is complete and correct. If you have cited papers that have been retracted, please include the rationale for doing so in the manuscript text, or remove these references and replace them with relevant current references. Any changes to the reference list should be mentioned in the rebuttal letter that accompanies your revised manuscript. If you need to cite a retracted article, indicate the article's retracted status in the References list and also include a citation and full reference for the retraction notice.

Response: The reference list has been reviewed to ensure that it is complete and correct. None of the original references was retracted, and a few additional references were added relevant to the data analysis write up in the methods section.

Reviewers' comments:

Reviewer's Responses to Questions

Comments to the Author

1. Does this manuscript meet PLOS Global Public Health's publication criteria? Is the manuscript technically sound, and do the data support the conclusions? The manuscript must describe methodologically and ethically rigorous research with conclusions that are appropriately drawn based on the data presented.

Reviewer #1: Yes Reviewer #2: Yes

---

2. Has the statistical analysis been performed appropriately and rigorously?

Reviewer #1: Yes Reviewer #2: Yes

---

3. Have the authors made all data underlying the findings in their manuscript fully available (please refer to the Data Availability Statement at the start of the manuscript PDF file)?

The PLOS Data policy requires authors to make all data underlying the findings described in their manuscript fully available without restriction, with rare exception. The *data should be provided as part of the manuscript or its supporting information, or deposited to a public repository*. For example, in addition to summary statistics, the data points behind means, medians and variance measures should be available. If there are restrictions on publicly sharing data—e.g. participant privacy or use of data from a third party—those must be specified.

Reviewer #1: No Reviewer #2: Yes

Response: We have submitted our data to the journal alongside the revised manuscript.

---

4. Is the manuscript presented in an intelligible fashion and written in standard English?

PLOS Global Public Health does not copyedit accepted manuscripts, so the language in submitted articles must be clear, correct, and unambiguous. Any typographical or grammatical errors should be corrected at revision, so please note any specific errors here.

Reviewer #1: Yes Reviewer #2: Yes

---

5. Review Comments to the Author

Please use the space provided to explain your answers to the questions above. You may also include additional comments for the author, including concerns about dual publication, research ethics, or publication ethics. (Please upload your review as an attachment if it exceeds 20,000 characters)

Reviewer #1:

Title: Magnitude and factors associated with post-tuberculosis lung disease in low and middle-income settings: a systematic review and meta-analysis
• Is it settings or countries?

Response: This inconsistency is noted. The correct term is "countries", the title has been revised accordingly to replace "settings" with "countries". This has been applied across the manuscript.

Abstract:
• The objective is merged with the methods in the abstract. It could be better to move it to the background with the justifications of conducting this review.

Response: This revision has been effected in the abstract.

• Is it lower or low income countries? Please use the appropriate word consistently throughout the manuscript

Response: We have corrected this and used the term "low-income countries" throughout the manuscript.

• Statistical methods and the software used to compute the magnitude are not described. Try to describe some of the statistical methods and the selected effect sizes in methods part.

Response: We have added a statement in the methods section of the abstract to briefly describe the software and statistical analysis. To remain within the word limits for the abstract we kept the description brief. The more detailed description is provided in the full text of the paper.

• Details of characteristics of included studies were included in the results. Focus on the main findings addressing the objectives.

Response: We have reduced the number of study characteristics in results of the abstract to the key characteristics of studies.

• Keywords are too much. Please reduce them to the recommended.

Response: The PLOS author guidelines online give no limitation to the number of allowable keywords, however I may have mis-understood the guidance and stand to be corrected. We have reduced the number of keywords has been reduced as requested, we hope that they are now within the desirable limit.

"post-TB lung disease; chronic lung disease; tuberculosis sequelae; lung function; spirometry; respiratory symptoms; radiology; HIV; sub-Saharan Africa; LMIC."

Introduction:

• Paragraph 1: TB can be also caused by other species of Mycobacterium and if you didn't use terms that can exclude them, it will be confusing. So it may be better to use "usually caused by MTB" or "mostly caused by MTB".

Response: My understanding has been that the disease tuberculosis is caused only by Mycobacterium tuberculosis. Infection or disease caused by non-tuberculous mycobacteria (NTM) such as M. kansasi, M. avium intracellulaire etc are termed as "non-tuberculous mycobacterial infections" or infections by "mycobacteria other than tuberculosis – MOTT" and are not considered to be "tuberculosis".

We therefore prefer not to alter the phrasing if this may be acceptable to the reviewer.

Methods:

• Literature search: the search terms used are narrow and missed important key term tuberculosis.

Response:

The total of fifty (50) search terms were used. We attempted to include adequate key search terms for the various key variables of interest per our study objectives. Supporting table 1 provides full details of the search terms that were included.

The researchers deliberated carefully about the appropriate search terms to include to ensure the search terms are broad enough to capture the available literature on the outcome of interest. We also noted that the term "tuberculosis" on its own is widely used to refer to active TB disease, and searches that include "tuberculosis" as a key term return hundreds of thousands of publications on TB as a disease.

*After these considerations, in order to capture post-TB papers as opposed to "active TB" the authors deliberately used key words that included the word "tuberculosis" OR "TB" together with a term relevant to the outcome of interest of lung disease or damage AFTER TUBERCULOSIS (e.g. "TB sequelae" or "PTLD") per the international consensus paper recommendation for research on the subject (Allwood et al, 2020). The word TB or tuberculosis appeared **eight times** among the search*

terms, and we deemed this adequate to capture most literature pertinent to the outcome of interest, indeed the search returned an initial 3,934 records (figure 1).

• Eligibility criteria: you stated that only articles written in English, or those with an English translation were eligible for inclusion; but you have described above in the literature search that no language restrictions was not imposed. How this contrasting information should be contextualized? If there are studies published in languages other than English, would you require translations or will you (the reviewers) translate them?

Response:

At initial search, no language restrictions were imposed to ensure that we did not miss studies published in other languages that may have English translations. At abstract screening, two reviewers identified those studies in other languages that had English translations and were able to assess them for eligibility for the review. Those that had no English translation available were excluded. The reviewers did not translate any studies into English themselves. (We did not have capability or the resources to translate any articles ourselves.)

We removed the sentence from the section in which the search strategy was described since language selection was not among the search terms, and retained the statement on inclusion / exclusion criteria in the section on eligibility criteria. We hope that this makes our process clearer.

• Study selection: what quality of studies will be included? What is the importance of evaluating the quality of studies using Newcastle-Ottawa scale if poor quality studies are also included in your review? Is any quality of study with outcome of interest be selected? Is any sample size of studies be included? How many study participants are required from a single study to be included in your review? Brief description of these variables are required.

Response: Study quality was not an inclusion or exclusion criteria. We aimed to report all eligible evidence on post-TB lung disease regardless of study quality. The purpose of evaluating methodological quality was to provide insight on the quality of evidence available on post-TB lung disease, and to enable this to contribute to interpretation of findings. Any sample size of study was considered eligible for inclusion as long as the study provided key outcome data on prevalence of PTLD. Case reports and case series were excluded as by design they do not provide prevalence data, they report only patients with the disease of interest.

We have articulated this information in the revised paper.

• Statistical analysis: which effect sizes are used to identify factors associated with PTLD?

Response:

The factors of interest in these meta-analyses were largely whole study characteristics such as geographical region, low- vs middle-income setting, urban vs rural setting, and adult study population vs child study population. To evaluate factors associated with PTLD, studies were stratified (grouped) by the factor of interest (e.g. studies in Africa, SE Asia, etc), then random effects meta-analysis was performed and Cochran's Q test applied to compare pooled prevalence of the outcome (PTLD) between subgroups or strata for each factor of interest, and significance was defined at p value <0.05.

Effect sizes such as odds ratios for a specific risk factor at patient level requires each study to have a mix of "exposed" and "unexposed" for the specific factor, and to provide odds ratio for outcome comparing exposed to unexposed.

We examined each paper for reported odds ratios, relative risks and for disaggregated outcome data for the following patient specific factors: HIV+ vs HIV- status, male vs female, child vs adult disaggregated for the outcome of interest. Few studies (<4 out of 30 studies) computed odds ratio or disaggregated patient level data for any of these three patient level characteristics. The majority of papers only provided information on these factors in their initial description of characteristics of study participants, (e.g % of whole study population that were female) but did not compute odds ratios by these factors, neither did they disaggregate these data by risk factor status when reporting PTLD outcomes (i.e. % of females who had PTLD, % of males who had PTLD).

The following references have been added in the methods write-up to strengthen the explanation of data analytic approaches used in the paper:

> *Harrer, M., Cuijpers, P., Furukawa, T.A., & Ebert, D.D. (2021). Doing Meta-Analysis with R: A Hands-On Guide (1st ed.). Chapman and Hall/CRC. https://doi.org/10.1201/9781003107347*
>
> *Balduzzi S, Rücker G, Schwarzer G. How to perform a meta-analysis with R: a practical tutorial. Evid Based Ment Health. 2019 Nov;22(4):153-160. doi: 10.1136/ebmental-2019-300117. Epub 2019 Sep 28. PMID: 31563865.*

Results:
• Eventually identified 102 eligible studies and subjected these to full paper review for eligibility. What does it mean? Have you identified 102 studies for eligibility or inclusion? It needs revision.

Response: We appreciate the comment. The correct statement is as follows:

At abstract screening we identified 102 "potentially eligible" studies and subjected them to full paper assessment for eligibility. We have reworded this statement in the manuscript.

• 18 studies were excluded from your study because they are from high income countries. How it comes to full article review as your eligibility criteria stated that

patients must have been living and studies conducted from low or middle income countries?

Response: These 18 studies did not describe the study location/country or study site in the abstract, and this information was available in the full paper content. For this reason, it was established when reading the full text of the paper that the study was conducted in a high-income country. The findings from those studies were referred to in discussion to contrast with findings from LMICs.

• Description of studies: para-2: The sample size was expressed in “subjects” but it is commonly advised to use “participants” or “patients” in human studies.

Response: The term “subjects” has been expressed as “participants” or “patients” as appropriate throughout the manuscript.

• Table 1b: what is the importance of expressing gender in terms of female (%)?

Response: Perhaps the more appropriate heading would be “sex”, and the reported percentage in this column is “% female”. i.e. what proportion of the study population are female. The column heading has been revised accordingly to read “Sex (% female)”.

Discussion:

• The review and meta-analysis quantitatively reveal a high burden of post-TB lung disease among persons living in lower and lower-middle income settings. This lacks consistency of using LMIC. Are they “lower and lower middle” or “low and middle income?” Use these terms consistently.

Response: This comment is appreciated. The text has been revised to state “low- and middle-income countries” as appropriate in the manuscript.

Reviewer #2:

Obimbo et al provide an excellent systematic review and metanalysis of studies that provided data on the magnitude (incidence /prevalence) of post TB lung disease in low- and middle-income countries. Data obtained from 32 studies and involving 6,050 patients revealed high rates of lung function impairment (pooled prevalence 46.7%, 95% CI 36.9-56.7%, from 2,701 patients who had this information), persistent symptoms (pooled prevalence 41.0,% 95% CI 29.39-53.70% , from 3, 811 patients) and radiological abnormalities (pooled prevalence 64.63%, 95% CI 39.54 -83.63%, from 2,857 patients). The presence of any one of these measures of PTLD was found in 42.7% of the 6,050 patients (95% CI 34.64-51.14.)

There were regional differences in the magnitude of PTLD with the WHO regions of Western Pacific and Southeast Asia having the highest rates. Risk factor analysis revealed living or receiving treatment from a rural setting, being HIV seronegative and being older to be associated with higher rates of PTLD. There were only two studies that provided information on PTLD following treatment of multidrug resistant TB, both of which showed high rates of post TB sequelae more than 90% of patients.

The paper is well written. There are only minor issues that the authors may want to address:

1. In the introduction section it is stated that there were 10 million people who were diagnosed to have TB in 2019 of whom 1.2 million died of the disease. This is an erroneous interpretation of the information provided by the WHO global TB report. The figures provided in the WHO global TB report are estimates of the global incidence of TB (10 million people in 2019) and global mortality (1.2 million people) from the disease. In 2019, only about 7 million people were diagnosed to have TB and were reported to national TB programs and consequently reported to WHO leaving a TB notification gap of about 3 million people. The deaths from TB in that year are computed from both the notified and unnotified groups.

Response: This is noted, your comment is appreciated. The statement has been revised to reflect that these are estimates.

2. It may be good to present the data on incidence and prevalence separately. These two epidemiologic parameters provide significantly different information with different implications for programming for post TB care.

Response: None of the studies reported incidence data, all studies reported outcome as prevalence (as percent), or proportion of individuals with PTLD after completing TB treatment, which was computed from reported numbers with damaged lungs divided by total number of individuals who completed TB treatment. We were also surprised at this finding. However after further thought, perhaps this is explainable due to the clinical evolution of lung injury during active TB disease - the lung damage (outcome) has already occurred by the time the patient completes TB treatment, and

in the period thereafter no new incident injury may be attributable to the TB after the fact.

3. I am wondering why statistical measures of association (odds ratios or relative risk) are not provided for the risk factor analysis for PTLD.

Response:

The factors of interest in these meta-analyses were largely whole study characteristics such as geographical region, lower vs middle income setting, urban vs rural setting, and adult study population vs child study population. Random effects meta-analysis was done and Cochran's Q test applied to evaluate for differences in prevalence between individual studies, and compare pooled prevalence between strata (or subgroups).

Effect sizes such as odds ratios and 95% confidence intervals for the O.R. for a specific risk factor at patient level requires each study to provide odds ratio for outcome of interest among those with or without the specific risk factor (such as HIV status or sex, or patient age), OR provide disaggregated data on each patient's exposure / non exposure by the risk factor.

We examined each paper for reported odds ratios, relative risks and for disaggregated outcome data for the following patient specific factors: HIV+ vs HIV- status, male vs female, child vs adult disaggregated for the outcome of interest. Few studies (<5 out of 30 studies) computed odds ratio or disaggregated patient level data for any of these three patient level characteristics. The majority of papers only provided information on these factors in their initial description of characteristics of study participants, (e.g % of whole study population that were female) but did not disaggregate these data by risk factor status when reporting PTLD outcomes (i.e. % of females who had PTLD, % of males who had PTLD). For HIV status specifically, only 4 studies had a mix of HIV+ and HIV- patients, and 2 of these reported odds ratio for PTLD by HIV status, the other 2 studies did not. Most studies had purely HIV+ patients, or purely HIV- patients where HIV status was specified. There was therefore inadequate number of studies reporting OR or with disaggregated patient level data to enable a pooled odds ratio that is representative to be computed.

4. I note that there are a few sentences on page 29 that are have grammatical errors.

Response: This is noted, the errors have been corrected.
