# Supplementary material for: Magnitude and factors associated with post-tuberculosis lung disease in low- and middle-income countries: A systematic review and meta-analysis
Source: PLOS Glob Public Health. 2022 Dec 20;2(12):e0000805. doi: 10.1371/journal.pgph.0000805 (PMC10021795; doi:10.1371/journal.pgph.0000805)
Supplement: Attachment — Submitted filename: Response to Reviewers.docx [file pgph.0000805.s008.docx]

**UNIVERSITY OF NAIROBI**

**FACULTY OF HEALTH SCIENCES**

# SCHOOL OF MEDICINE

# DEPARTMENT OF PAEDIATRICS AND CHILD HEALTH

Kenyatta National Hospital

Telephone: +(254) 20 4915046	P.O. Box 19676

Email : [dept-paediatrics@uonbi.ac.ke](mailto:dept-paediatrics@uonbi.ac.ke)	Nairobi, Kenya. 00202

To:

The Academic Editor,

PLoS Global Public Health

31^st^ August 2022

Dear Ms Lucy Chimoi,

**RE: Response to Reviewers: Magnitude and factors associated with post-tuberculosis lung disease in low- and middle-income settings: a systematic review and meta-analysis (PGPH-D-22-01006)**

Thank you very much for reviewing our above-mentioned systematic review and for giving us an opportunity to address the reviewers’ comments. The comments were helpful and valuable to improve and refine the manuscript. We have revised the manuscript accordingly and submit both track changes and clean versions of the revised manuscript. We have made a point-by-point response to the comments of both the editor and the reviewers, each response is inserted below the specific comment. All the required documents have been submitted using the online system.

We do hope that we have responded adequately to the comments of the reviewers, and that we have addressed all the requirements of the editors and journal. Once again, we thank PLOS Global Public Health and the reviewers for your timely review of this systematic review and meta-analysis on post-tuberculous lung disease in low- and middle-income countries.

We look forward to hearing from you.

Yours Sincerely,

Elizabeth Maleche-Obimbo

Professor of Paediatrics & Respiratory Medicine

Epidemiologist and Paediatric Pulmonologist

Department of Paediatrics & Child Health

Faculty of Medicine, University of Nairobi.

Email: [Elizabeth.obimbo@uonbi.ac.ke](mailto:Elizabeth.obimbo@uonbi.ac.ke)

**Response to Reviewers Comments:**

**Journal Requirements:**

1. Please ensure that the Title in your manuscript file and the Title provided in your online submission form are the same.

*Response: We note the omission of the dash in the title. We have ensured that the title in the manuscript is the same as that in the online submission form.*

2. Please review your reference list to ensure that it is complete and correct. If you have cited papers that have been retracted, please include the rationale for doing so in the manuscript text, or remove these references and replace them with relevant current references. Any changes to the reference list should be mentioned in the rebuttal letter that accompanies your revised manuscript. If you need to cite a retracted article, indicate the article’s retracted status in the References list and also include a citation and full reference for the retraction notice.

*Response: We have reviewed our reference list and ensured that it is complete and correct. No papers have been retracted and no changes were made to the reference list.*

3. We ask that a manuscript source file is provided at Revision. Please upload your manuscript file as a .doc, .docx, or .rtf.

*Response: We have submitted a marked-up copy of the manuscript that highlights changes made, and also submitted a clean version.*

4. Please update your *Supporting Information list of legends to include all supplementary files.*

*Response: We have updated the supporting information list of legends to include all supplementary files.*

**Reviewers' comments:**

Reviewer's Responses to Questions

**Comments to the Author**

1. Reviewer #1: Thank you for your response to almost all my comments.

Introduction:
• Paragraph 1: TB can be also caused by other species of Mycobacterium and if you didn’t use terms that can exclude them, it will be confusing. So it may be better to use “usually caused by MTB” or “mostly caused by MTB”.

But the authors response to my one comment on the cause of TB is not yet well addressed. You said that TB is caused by only MTB. But the literature and other relevant credible sources described that TB is caused by M. tuberculosis complex that comprised of MTB, M. bovis, M. africanum and other related species.The mycobacterial species that occurs in human and belong to M. tuberculosis complex include M. tuberculosis, M. bovis, M. bovis BCG and M. africanum: all species are capable of causing tuberculosis. You can check at <https://www.nationaljewish.org/conditions/tuberculosis-tb/causes>. As the authors didn't specifically describe the disease caused is a type of pulmonary TB, it will be difficult to agree with and it is misleading to declare TB is only caused by MTB. Or the authors should describe it as "post-pulmonary tuberculosis" in the title or elsewhere in the mansucript.

*Response: Thank you for this comment, and the literature that you have shared was very informative. We now understand that the other mycobacteria are termed to be part of M tuberculosis complex. We have revised the statement in the introduction as guided to read “tuberculosis is mostly caused by Mycobacterium tuberculosis”.*

_______________________________________________________________________
