# Supplementary material for: Magnitude and factors associated with post-tuberculosis lung disease in low- and middle-income countries: A systematic review and meta-analysis
Source: PLOS Glob Public Health. 2022 Dec 20;2(12):e0000805. doi: 10.1371/journal.pgph.0000805 (PMC10021795; doi:10.1371/journal.pgph.0000805)
Supplement: Attachment — Submitted filename: Response to Reviewers_25-Oct-22 (1).docx [file pgph.0000805.s009.docx]

**UNIVERSITY OF NAIROBI**

**FACULTY OF HEALTH SCIENCES**

# SCHOOL OF MEDICINE

# DEPARTMENT OF PAEDIATRICS AND CHILD HEALTH

Kenyatta National Hospital

Telephone: +(254) 20 4915046	P.O. Box 19676

Email : [dept-paediatrics@uonbi.ac.ke](mailto:dept-paediatrics@uonbi.ac.ke)	Nairobi, Kenya. 00202

To:

The Academic Editor,

PLoS Global Public Health

25^th^ October 2022

Dear Ms Lucy Chimoi,

**RE: Response to Reviewers: Magnitude and factors associated with post-tuberculosis lung disease in low- and middle-income settings: a systematic review and meta-analysis (PGPH-D-22-01006)**

Thank you very much for reviewing our above-mentioned systematic review and for giving us an opportunity to address the reviewers’ comments. The comments were helpful and valuable to improve and refine the manuscript. We have revised the manuscript accordingly and submit both track changes and clean versions of the revised manuscript. We have made a point-by-point response to the comments of both the editor and the reviewers, each response is inserted below the specific comment. All the required documents have been submitted using the online system.

We do hope that we have responded adequately to the comments of the reviewers, and that we have addressed all the requirements of the editors and journal. Once again, we thank PLOS Global Public Health and the reviewers for your timely review of this systematic review and meta-analysis on post-tuberculous lung disease in low- and middle-income countries.

We look forward to hearing from you.

Yours Sincerely,

Elizabeth Maleche-Obimbo

Professor of Paediatrics & Respiratory Medicine

Epidemiologist and Paediatric Pulmonologist

Department of Paediatrics & Child Health

University of Nairobi.

Email: [Elizabeth.obimbo@uonbi.ac.ke](mailto:Elizabeth.obimbo@uonbi.ac.ke)

**Response to Reviewers Comments:**

**Editor Comments:**

1. Please review your reference list to ensure that it is complete and correct. If you have cited papers that have been retracted, please include the rationale for doing so in the manuscript text, or remove these references and replace them with relevant current references. Any changes to the reference list should be mentioned in the rebuttal letter that accompanies your revised manuscript. If you need to cite a retracted article, indicate the article’s retracted status in the References list and also include a citation and full reference for the retraction notice.

*Response:*

*We added one additional paper by Migliori et al in the introduction section as requested by Reviewer* #*. The added paper appears as reference no. 13.*

*We also identified a second highly relevant paper published in recent weeks by Nkereuwem E et al that examined impaired lung function in children with prior TB in The Gambia. We took the opportunity to add this in the relevant paragraph of the discussion. The added paper is reference no. 61.*

*The numbering of all cited papers was revised accordingly. No papers were retracted from the reference list. We have reviewed our reference list and ensured that it is complete and correct. The additional reference citations are hereby provided.*

Migliori GB, Marx FM, Ambrosino N, Zampogna E, Schaaf HS, van der Zalm MM, et al. Clinical standards for the assessment, management and rehabilitation of post-TB lung disease. Int J Tuberc Lung Dis. 2021;25(10):797-813. doi: 10.5588/ijtld.21.0425. PubMed PMID: 34615577; PubMed Central PMCID: PMCPMC8504493.

Nkereuwem E, Agbla S, Sallahdeen A, Owolabi O, Sillah AK, Genekah M, Tunkara A, Kandeh S, Jawara M, Saidy L, Bush A, Togun T, Kampmann B. Reduced lung function and health-related quality of life after treatment for pulmonary tuberculosis in Gambian children: a cross-sectional comparative study. Thorax. 2022 Sep 15:thoraxjnl-2022-219085. doi: 10.1136/thorax-2022-219085. Epub ahead of print. PMID: 36109164.

2. Thank you for revising your manuscript. Besides the reviewer suggestion, please include in your discussion an explanation of why you decided to pool studies despite the high heterogeneity.

*Response: We have added a statement in the discussion section on the same as follows:*

*“There was significant heterogeneity for the primary outcome (prevalence of PTLD), however this was an expected finding given the heterogeneity across included studies with regards to study setting, access to health care, study population characteristics, and regional differences in TB epidemiology. We therefore applied a random effects model in the meta-analyses to account for heterogeneity and assessed residual heterogeneity using sensitivity analyses.”*

3. In your next revised version, please ensure that you have edited and minutiously reviewed the entire text, as some minor editorial issues remain (final dot missing at the end of sentences, incomplete sentence page 26 and others).

*Response: We have reviewed the entire manuscript and corrected any typographical and grammatical errors therein.*

**Reviewers' comments:**

Reviewer's Responses to Questions

**Comments to the Author**

1. Reviewer #3

"After the first revisions, the manuscript improved significantly. However, there is an important recent document that should be cited and discussed by the authors:
Migliori GB et al. Clinical standards for the assessment, management and rehabilitation of post-TB lung disease. IJTLD 2021; 25(10):797-813. doi: 10.5588/ijtld.21.0425.

Response: *Thank you for bringing this highly relevant publication to our attention. We have added a relevant statement in the introduction and cited the Migliori paper. The added paper appears as reference no. 13.*

_______________________________________________________________________
